# Metabolome analysis of *Saccharomyces cerevisiae* and optimization of culture medium for *S*-adenosyl-l-methionine production

**DOI:** 10.1186/s13568-016-0210-3

**Published:** 2016-06-09

**Authors:** Kenshi Hayakawa, Fumio Matsuda, Hiroshi Shimizu

**Affiliations:** Department of Bioinformatic Engineering, Graduate School of Information Science and Technology, Osaka University, 1-5 Yamadaoka, Suita, Osaka, 565-0871 Japan; KANEKA Fundamental Technology Research Alliance Laboratories, Graduate School of Engineering, Osaka University, 2-8 Yamadaoka, Suita, Osaka, 565-0871 Japan

**Keywords:** Metabolome analysis, Central carbon metabolism, *Saccharomyces cerevisiae*, *S*-adenosyl-l-methionine, Fermentation engineering

## Abstract

*S*-Adenosyl-l-methionine (SAM) is a fine chemical used as a nutritional supplement and a prescription drug. It is industrially produced using *Saccharomyces cerevisiae* owing to its high SAM content. To investigate the optimization of culture medium components for higher SAM production, metabolome analysis was conducted to compare the intracellular metabolite concentrations between Kyokai no. 6 (high SAM-producing) and laboratory yeast S288C (control) under different SAM production conditions. Metabolome analysis and the result of principal component analysis showed that the rate-limiting step for SAM production was ATP supply and the levels of degradation products of adenosine nucleotides were higher in Kyokai 6 strain than in the S288C strain under the l-methionine supplemented condition. Analysis of ATP accumulation showed that the levels of intracellular ATP in the Kyokai 6 strain were also higher compared to those in the S288C strain. Furthermore, as expected from metabolome analysis, the SAM content of Kyokai 6 strain cultivated in the medium without yeast extract increased by 2.5-fold compared to that in the additional condition, by increasing intracellular ATP level with inhibited cell growth. These results suggest that high SAM production is attributed to the enhanced ATP supply with l-methionine condition and high efficiency of intracellular ATP consumption.

**Electronic supplementary material:**

The online version of this article (doi:10.1186/s13568-016-0210-3) contains supplementary material, which is available to authorized users.

## Introduction

**Fig. 1:**
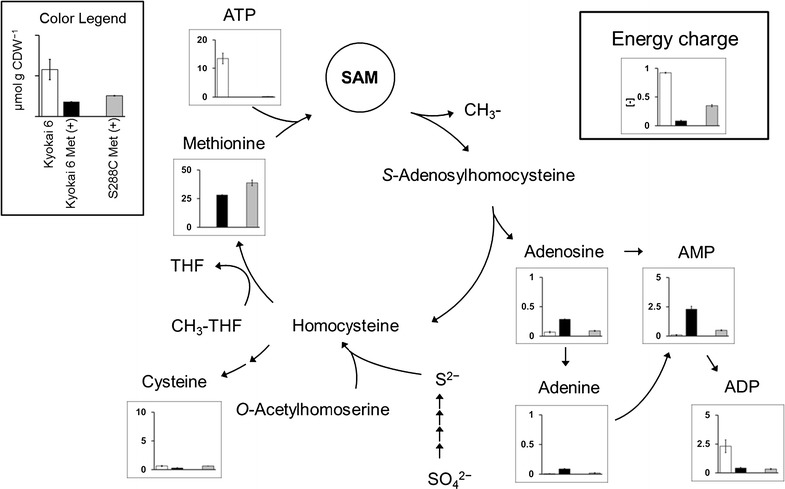
Methionine salvage pathway for SAM biosynthesis in *S. cerevisiae* and metabolome data of these pathway metabolites obtained by CE-TOFMS. *White bars* represent Kyokai 6 strain without additional l-methionine. *Black* and *gray bars* represent Kyokai 6 strain and S288C strain in additional l-methionine, respectively. Values except energy charge on the *y-axis* in each* graph* represent the concentrations (μmol g CDW^−1^) of metabolites, the average of triplicate experiments, with *error bars* calculated as the standard deviations of the means. *THF* tetrahydrofolate

*S*-Adenosyl-l-methionine (SAM) is a commercialized fine chemical used as a nutritional supplement and a prescription drug. *Saccharomyces cerevisiae* is used as an industrial production host owing to the SAM accumulation capability of the Japanese sake brewing strains (Shiozaki et al. 1984). SAM is synthesized from methionine and ATP by methionine adenosyltransferase in *S. cerevisiae*. After acting as a biological methyl group donor, methionine is regenerated from *S*-adenosylhomocysteine via the methionine salvage pathway (Fig. 1). The intracellular concentration of SAM has been increased by a strain improvement based on mutagenesis. The concentration of SAM was improved 20-fold greater than that produced by the control *S. cerevisiae* strain by transforming ethionine resistance gene (Shiomi et al. 1990). Nystatin-resistant mutants with insufficient ergosterol biosynthesis produced threefold SAM greater than their parent strains and accumulated 70 mg (g cell dry weight [CDW])^−1^ (Shobayashi et al. 2006). The SAM accumulation reached 18 mg g CDW^−1^ by a disruption of the adenosine kinase (*ADO1*) gene in the methionine salvage pathway (Kanai et al. 2013).

Another improvement strategy is an optimization of culture conditions. For example, the addition of methionine to the medium was effective for SAM production (Shiozaki et al. 1984; Shobayashi et al. 2007a). ^13^C-metabolic flux analysis also suggested that aeration is an important factor since enhanced ATP regeneration with high TCA cycle flux was related to high SAM production in Japanese sake brewing strains (Hayakawa et al. 2015).

In the present study, a strategy for further improvement of SAM production was investigated by comparison of the metabolite accumulation profiles of *S. cerevisiae* among various SAM productivity conditions. For this purpose, metabolome analysis using capillary electrophoresis time-of-flight mass spectrometry (CE-TOFMS) was employed for the comprehensive analysis of intracellular metabolites including sugar phosphates, nucleotides, amino acids, and organic acids (Ohashi et al. 2008; Soga et al. 2006). Metabolome analysis using CE-TOFMS has been employed previously to characterize intracellular metabolic states in *S. cerevisiae* (Hasunuma et al. 2011; Nugroho et al. 2015; Oldiges et al. 2007; Shirai et al. 2013). The metabolome analysis of *S. cerevisiae* showed that active SAM production causes a deficiency of ATP and an accumulation of the degradation products (AMP, adenine, and adenosine). Based on this finding, the culture medium was modified to increase intracellular ATP concentration by cell growth inhibition. The SAM concentration of Kyokai no. 6 strain was increased 2.5-fold on excluding the yeast extract from the culture medium.

## Materials and methods

### Strains and growth conditions

*Saccharomyces cerevisiae* strains, S288C (NBRC1136) and Kyokai no. 6 (NBRC2346), were purchased from the National Biological Resource Center (NBRC, Chiba, Japan). For the preculture, the stock cells were cultivated for 24 h at 30 °C with shaking in 4 mL medium (pH 6.2) containing 50.0 g L^−1^d-glucose, 10.0 g L^−1^ peptone (Nihon pharmaceutical, Tokyo, Japan), 5.0 g L^−1^ yeast extract (Bacto Yeast Extract, Difco Laboratories, Franklin Lakes, NJ, USA), 4.0 g L^−1^ KH_2_PO_4_, 2.0 g L^−1^ K_2_HPO_4_, 0.6 g L^−1^ MgSO_4_·7H_2_O. For SAM production assay, 1 % of preculture broth was inoculated in 4 mL SAM production medium, which contained the components described as above with or without 1.5 g L^−1^l-methionine (Hayakawa et al. 2015). All cultivations were performed for 24 h at 30 °C in triplicate.

### Off-line measurement

The analysis of SAM, CDW, glucose, and ethanol were performed as described by Hayakawa et al. (2015). SAM concentration was measured by high-performance liquid chromatography LC2010A-HT (Shimadzu, Kyoto, Japan) after 10 % HClO_4_ extraction. CDW was estimated using 1 OD_600_ corresponding to 0.21 g CDW L^−1^ in Kyokai 6 and 0.22 g CDW L^−1^ in S288C. Glucose and ethanol concentrations were measured enzymatically by using an analyzer (BF-7, Oji Scientific Instruments, Hyogo, Japan). The intracellular ATP concentration was measured as follows. Cultivated cells were harvested by filtration and washed with Milli-Q water twice. The membrane was frozen using liquid N_2_, and lyophilized. Dried cells were suspended in 1 mL Milli-Q water and boiled for 10 min at 95 °C. The suspension was centrifuged to collect the supernatant (Ando et al. 2006). The ATP contents in the supernatant were measured using Fluorometric and Colorimetric ATP Quantitation Kit (PromoCell, Heidelberg, Germany) and a spectrometer.

### Metabolome analysis

Intracellular metabolite concentrations were measured using CE-TOFMS, according to previously described methods (Nugroho et al. 2015; Yoshikawa et al. 2013). Cultivated cells were harvested by filtration and washed with Milli-Q water. After the membrane was soaked in methanol solution containing an internal standard (H3304-1002, Human Metabolome Technologies, Yamagata, Japan), ultra-sonication was performed. After removal of the membrane, chloroform and water were added to the solution. The aqueous portion collected after mixing with a vortex mixer was filtered by UltrafreeMC-PLHCC 250 (Human Metabolome Technologies) and dried. For CE-TOFMS, pellets were suspended in the second internal standard (H3304-1004, Human Metabolome Technologies) solution. Samples were analyzed using Agilent 7100 CE system with Agilent 6224 TOF–MS (Agilent Technologies, Santa Clara, CA, USA). Metabolome data were normalized and analyzed by PCA using Mass Profiler Professional (Agilent Technologies).

## Results

### Effect of cultivation condition on SAM production levels

Methionine and ATP are precursors for SAM biosynthesis in *S. cerevisiae* (Fig. 1). It has been reported that SAM contents in *S. cerevisiae* strains were increased by 1.5 g L^−1^ of l-methionine supplementation (Shiozaki et al. 1984; Shobayashi et al. 2007a), suggesting that the methionine supply is a bottleneck in SAM biosynthesis. In order to confirm the effects of methionine on SAM production, the control (laboratory strain S288C) and high SAM-producing (Kyokai 6 used for brewing Japanese sake) strains were cultured in media with or without 1.5 g L^−1^l-methionine. Cells were harvested upon the depletion of glucose in the broth (24 h). The physiological parameters in all cultivations are shown in Table 1.

**Table 1:** Fermentation profiles of high-SAM-producing (Kyokai 6) and laboratory (S288C) strains in the presence of glucose with or without l-methionine

Strain	Kyokai 6		S288C	
l-Methionine	−	+	−	+
SAM production (mg L^−1^)	7.5 ± 0.4	177.6 ± 4.0	6.3 ± 0.1	62.6 ± 0.9
Biomass (g CDW L^−1^)	6.7 ± 0.04	5.6 ± 0.06	5.5 ± 0.05	5.0 ± 0.05
SAM content (mg g CDW^−1^)	1.1 ± 0.01	31.8 ± 1.0	1.1 ± 0.01	12.5 ± 0.2
Ethanol (g L^−1^)	17.8 ± 0.5	20.0 ± 0.1	19.5 ± 0.2	20.7 ± 0.1
pH	5.7 ± 0.02	5.3 ± 0.05	6.0 ± 0.03	5.4 ± 0.03

All data were obtained from the averages of three independent experiments ± SD

Comparison of the fermentation profile (Table 1) showed that the SAM content in Kyokai 6 strains cultured with l-methionine (31.8 mg g CDW^−1^) was 28-fold higher than that in strains cultivated without l-methionine (1.1 mg g CDW^−1^). The ethanol titer was also increased while the cell growth and the pH were decreased by methionine addition. The SAM content in the Kyokai 6 strain with methionine supplementation was 2.5-fold higher than that in the S288C strain (12.5 mg g CDW^−1^). These results suggested that an addition of methionine in the medium changed the intracellular metabolism to enhance ethanol and SAM production.

### Effects of l-methionine addition on intracellular metabolite profile

**Fig. 2:**
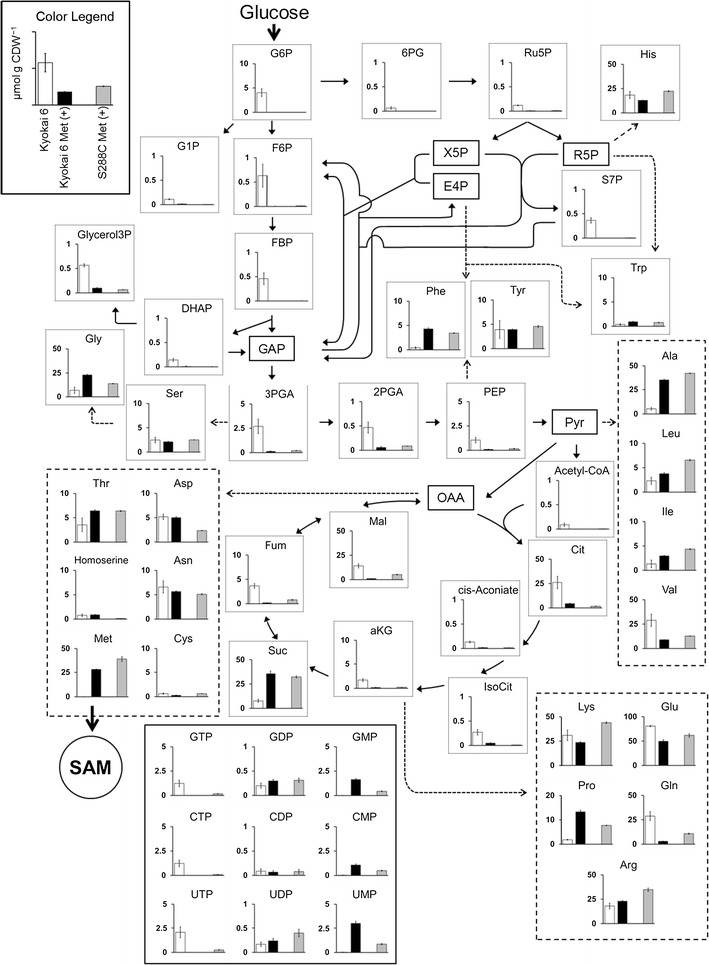
Metabolome data of central metabolic pathway metabolites obtained by the CE-TOFMS. *White bars* represent Kyokai 6 strain in no additional l-methionine. *Black* and *gray bars* represent Kyokai 6 strain and S288C strain in additional l-methionine, respectively. Values on the *y-axis* in each* graph* represent the concentrations (μmol g CDW^−1^) of metabolites, the average of triplicate experiments, with *error bars* calculated as the standard deviations of the means

The metabolic states under the two different SAM production conditions were compared by metabolome analysis. The metabolites were extracted from *S. cerevisiae* cells at 24 h after the cultivation start. The levels of 75 intracellular metabolites including amino acids, sugar phosphates, organic acids, and nucleotides were successfully determined by CE-TOFMS (Additional file 1: Table S1). The levels of metabolites in the methionine salvage pathway, glycolysis, pentose phosphate (PP) pathway, TCA cycle, cofactors, and amino acids are shown in Figs. 1 and 2, respectively.

The effects of methionine addition on the *S. cerevisiae* metabolome were investigated by a comparison between Kyokai 6 (white, without methionine addition) and Kyokai 6 Met(+) (black, with methionine addition) data, which is shown in Figs. 1 and 2. The metabolome data indicated that only a trace amount of methionine was observed in the Kyokai 6 strain without methionine addition (white, 0.06 μmol g CDW^−1^, Fig. 2). Since a relatively large amount of ATP was accumulated in the condition (13.6 μmol g CDW^−1^, Fig. 1), these results indicated that the l-methionine supply is a bottleneck in SAM biosynthesis.

The methionine depletion was solved in the Kyokai 6 Met(+), since the intracellular methionine concentration was increased more than 400× by the supplementation of 1.5 g L^−1^l-methionine in the medium (28.2 μmol g CDW^−1^, Fig. 2). In addition to the improvement of SAM production (Table 1), the metabolome data also revealed that the metabolic profile of the Kyokai 6 strain was drastically changed by methionine supplementation. For instance, while the succinate level was significantly increased, most intermediates in the glycolysis pathway, PP pathway, and TCA cycle were clearly decreased in Kyokai 6 Met(+) samples (black in Fig. 2) by methionine addition. These results suggested that the functions of the TCA cycle and oxidative phosphorylation were somehow inhibited by the excess methionine, being compensated by an activation of substrate-level phosphorylation with ethanol production (Table 1). This was supported by the intracellular concentrations of the nucleoside triphosphates including ATP and GTP (Figs. 1, 2). The metabolome data showed that intracellular ATP was depleted under the l-methionine supplemented condition (0.02 μmol g CDW^−1^, black in Fig. 1). Since the AMP level was increased, the adenylate energy charge (EC = ([ATP] + 0.5 × [ADP])/([ATP] + [ADP] + [AMP])) was decreased in Kyokai 6 Met(+) samples, indicating that the abilities for the anabolic and maintenance reactions were hampered by the methionine addition (Guimaraes and Londesborough 2008). These results suggested that ATP supply should be a rate-limiting step for SAM production in the methionine-supplemented condition (Fig. 1).

### Comparison between laboratory and SAM production strains

The SAM content in the Kyokai 6 strain was higher than that in the S288C strain with and without methionine supplementation (Table 1). The ^13^C-metabolic flux analysis performed in our previous study showed that active SAM biosynthesis in the Kyokai 6 strain should be derived from enhanced ATP regeneration with high TCA cycle flux (Hayakawa et al. 2015). It was also known that *S. cerevisiae* strains used for Japanese sake brewing showed high respiration activity and generally overexpress genes involved in the TCA cycle, respiration, and oxidative phosphorylation (Kasahara 1963; Shobayashi et al. 2007b). For further investigation of the effects of ATP levels on SAM productivity, intracellular ATP concentrations were determined by colorimetric analysis. The ATP level in Kyokai 6 strain (4.5 ± 0.4 μmol g CDW^−1^) was threefold higher than that in S288C strain (1.5 ± 0.06 μmol g CDW^−1^) without methionine supplementation. This result confirmed that Kyokai 6 strain has the potential for high SAM productivity due to high intracellular ATP level.

However, as mentioned above, the intracellular ATP was depleted in Kyokai 6 strain under the l-methionine supplemented condition (Fig. 1). It may indicate that the high ATP content could not contribute to the enhanced SAM production in Kyokai 6 strain. In order to address the problem, the metabolome data was additionally obtained from the laboratory yeast strain (S288C) cultivated with l-methionine (S288C Met(+) in Figs. 1, 2). The comparison between Kyokai 6 Met(+) (black) and S288C Met(+) (gray) data indicated that the metabolic profiles in the two conditions looked similar each other. To identify metabolites whose concentrations were changed in relation to SAM production, the principal component analysis (PCA) was performed using all data obtained from the three conditions.

**Fig. 3:**
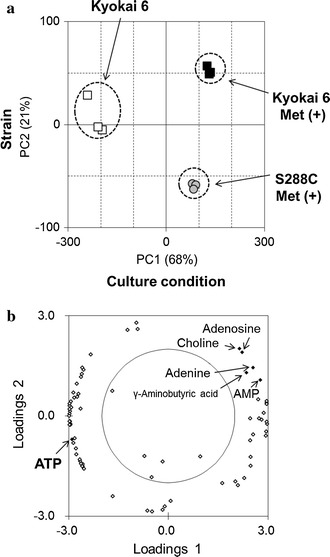
Score and factor loading plot of PCA in metabolome data. **a** PCA score plot of PC1 and PC2. *White squares* Kyokai 6 without l-methionine; *black squares* Kyokai 6 with l-methionine; *gray circles* S288C with l-methionine. **b** Factor loading plot for PC1 and PC2

The result of the PCA showed that the triplicate data of each condition formed distinct clusters (Fig. 3a). It can be observed that PC1 represents culture conditions with and without external supplementation of methionine (Fig. 3a). PC2 represents differences between Kyokai 6 and S288C strains. The factor loading scores indicated that AMP, adenine, adenosine, choline, and γ-aminobutyric acid positively contributed to the loadings of PC1 and PC2 (factor loadings scores were above 0.5, Fig. 3b; Additional file 1: Table S2). This indicates that the levels of AMP, adenine, and adenosine in Kyokai 6 Met(+) tended to be larger than that of S288C Met(+). Since these metabolites were degradation products of adenosine nucleotides derived from the methionine salvage pathway (Fig. 1), these results suggested that the methionine salvage pathway was actively working in the Kyokai 6 strain under the methionine-supplemented condition by a larger SAM supply.

### Effect of intracellular ATP concentration on SAM production

The metabolome analysis showed that the intracellular ATP was depleted in the methionine-supplemented condition, and suggested that the SAM production could be improved by the elevation of ATP supply. Since the supplementation of expensive materials such as ATP and the application of gene manipulation technologies are unpreferable for food production, a fermentation engineering technique was applied in this study by the optimization of culture medium composition.

**Fig. 4:**
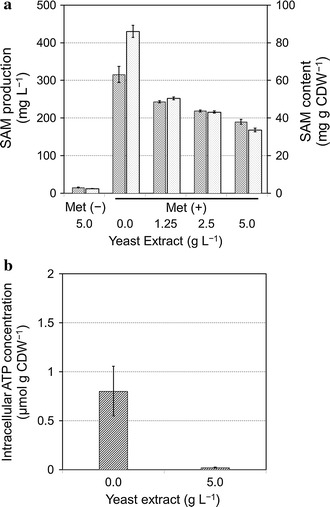
Effect of yeast extract content on the SAM production, SAM content, and ATP concentration in Kyokai 6 strain. **a** Effect of yeast extract content on the SAM production and SAM content. *Cross hatched* and *dotted bars* represent SAM production and SAM content, respectively. **b** Intracellular ATP concentration of Kyokai 6 strain without and with 5.0 g L^−1^ yeast extract obtained using CE-TOFMS. The *error bars* represent the standard deviations of the means

Yeast extract plays an important role for growth of *S. cerevisiae* cells since it consists of the water-soluble portion of autolyzed yeast, which contains amino acids, peptides, carbohydrates, vitamins and minerals (Jiang et al. 2010; Mosser et al. 2015; Thomas et al. 2002). To increase the ATP pool by reducing ATP demands for cell growth, the Kyokai 6 strain was cultivated in the medium containing various concentrations of yeast extract (0.0–5.0 g L^−1^). The results showed that the levels of both SAM production (mg L^−1^) and SAM content (mg g CDW^−1^) were increased whereas cell growth, ethanol production, and pH were decreased in response to the reduced yeast extract concentration (Additional file 1: Table S3). The maximal SAM production (315.6 mg L^−1^) and SAM content (86.1 mg g CDW^−1^) were observed using the yeast extract drop-out medium, whose levels were 1.6 and 2.5-fold higher than those in 5.0 g L^−1^ of yeast extract condition (189.8 mg L^−1^, 33.6 mg g CDW^−1^), respectively (Fig. 4a). It was also confirmed that SAM production was improved by the accumulation of intracellular ATP, since the concentration of ATP in the yeast extract drop-out condition (0.8 μmol g CDW^−1^) was about 40× higher than that in the 5.0 g L^−1^ yeast extract condition (0.02 μmol g CDW^−1^) (Fig. 4b).

## Discussion

**Fig. 5:**
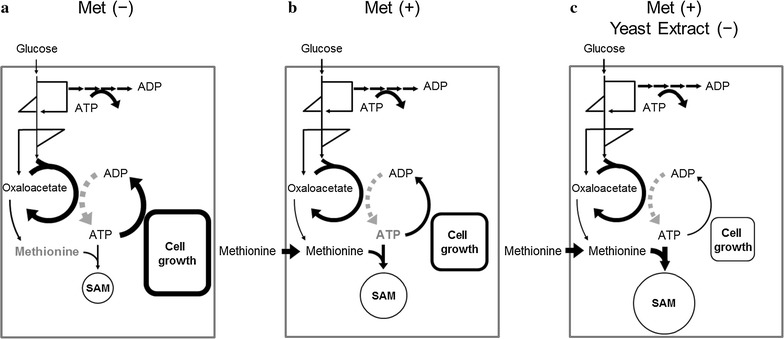
SAM production mechanism of high-SAM-producing strain estimated in this study. **a** Without methionine. **b** With methionine. **c** With methionine and without yeast extract. The *dotted gray arrows* represent respiration. The metabolome analysis results revealed that the rate-limiting step for SAM production without and with methionine was methionine and ATP supply, respectively (*gray characters*). Higher SAM production without yeast extract was explained by enhanced ATP supply with inhibited cell growth

In the present study, the intracellular metabolism of *S. cerevisiae* was compared in the various SAM production conditions by metabolome analysis using CE-TOFMS. The metabolome data showed that, whereas the methionine supply was a bottleneck in the SAM production of *S. cerevisiae* cells (Fig. 5a), ATP supply became insufficient under the methionine supplemented condition (Fig. 5b). Several studies of *S. cerevisiae* and other microorganisms such as *Escherichia coli* and *Candida utilis* supported the results of the metabolome analysis (Figs. 1, 2) since further improvements of SAM production were attained by an elevation of intracellular ATP level by excess oxygen supply, gene knockdown, and optimization of carbon source feeding (Chen et al. 2015; Li et al. 2012; Wang et al. 2013). It was reported that SAM accumulation in *S. cerevisiae* was increased by improving methionine and ATP availability by *MET6* and *SAM2* co-expression combined with sodium citrate feeding (Chen et al. 2016). The metabolome data also revealed that the levels of SAM degradation products such as AMP were increased under l-methionine supplemented condition (Figs. 1, 3b). Since these metabolites were produced via the methionine salvage pathway, it is expected that SAM production would be further enhanced by down-regulation of the pathway activity.

In order to increase ATP pool size, the culture medium composition was optimized in this study because gene manipulation technologies are not preferable for industrial food production. The restriction of cell growth, and increased intracellular ATP concentration and SAM production were achieved by reducing the supplemented yeast extract (Fig. 5c). The SAM concentration (86.1 mg g CDW^−1^) was larger than that reported previously (70 mg g CDW^−1^) (Shobayashi et al. 2006). On the other hand, the production of 10.8 g L^−1^ SAM was achieved by the cultivation of yeast for 5 days in a 10-L fermenter (Shiozaki et al. 1986) indicating that the volumetric productivity of SAM could be increased by improvement in intracellular SAM concentration and biomass levels. Thus, further improvements is required in the fermentation process such as introducing a two-phase process including a cell growth with rich medium and inhibited cell growth with restricted yeast extract. These results demonstrated that the metabolome analysis could offer useful information for productivity improvement in fermentation engineering. Furthermore, the reduction of the yeast extract is a cost-effective strategy for producing SAM and should be utilized to improve the production of other useful fine chemicals.

The results of this study uncovered that SAM content was increased by cell growth inhibition via an elevation of intracellular ATP level, whereas it has been reported that cell growth inhibition by lower agitation speed and feeding ethanol was effective to improve SAM content in *S. cerevisiae* (Shiozaki et al. 1989). The results suggested that the improved ATP supply enhanced the tolerance of *S. cerevisiae* against the lower pH conditions for uncontrolled pH cultivations and indirectly contributed to active SAM biosynthesis. This is because metabolite production was inhibited under the acid stress condition in *S. cerevisiae* (Sakihama et al. 2015), and intracellular ATP accumulation was related to acid stress tolerance of *S. cerevisiae* (Nugroho et al. 2015; Zhou et al. 2011). The strategy developed in this study would be useful to improve other bio-based productions involved in ATP supply such as glutathione and succinate (Hara and Kondo 2015). A novel methodology for more efficient SAM production would be addressed by further investigation of the metabolism and its relationship with the physiology of *S. cerevisiae.*

## Additional file

10.1186/s13568-016-0210-3 Metabolite concentration obtained by CE-TOFMS (μmol g CDW^−1^). **Table S2.** Factor loading corresponding to the PC1 and PC2. **Table S3.** Fermentation profiles of Kyokai 6 strains in the presence of glucose with 1.5 g L^−1^ methionine.
