# Supplementary material for: Metabolome analysis of Saccharomyces cerevisiae and optimization of culture medium for S-adenosyl-l-methionine production
Source: AMB Express. 2016 Jun 9;6:38. doi: 10.1186/s13568-016-0210-3 (PMC4899347; doi:10.1186/s13568-016-0210-3)
Supplement: Supplementary file 1 — 10.1186/s13568-016-0210-3 Metabolite concentration obtained by CE-TOFMS (μmol g CDW−1). Table S2. Factor loading corresponding to the PC1 and PC2. Table S3. Fermentation profiles of Kyokai 6 strains in the presence of glucose with 1.5 g L−1 methionine. [file 13568_2016_210_MOESM1_ESM.doc]

**AMB Express**

**Supporting information**

**Metabolome analysis of *Saccharomyces cerevisiae* and optimization of culture medium for *S*-adenosyl-l-methionine production**

Kenshi Hayakawa^1,2^, Fumio Matsuda^1^, Hiroshi Shimizu^1*^

^1^Department of Bioinformatic Engineering, Graduate School of Information Science and Technology, Osaka University, 1-5 Yamadaoka, Suita, Osaka 565-0871, Japan

^2^KANEKA Fundamental Technology Research Alliance Laboratories, Graduate School of Engineering, Osaka University, 2-8 Yamadaoka, Suita, Osaka, 565-0871, Japan

*Corresponding author

Hiroshi Shimizu

Department of Bioinformatic Engineering, Graduate School of Information Science and Technology, Osaka University, 1-5 Yamadaoka, Suita, Osaka, Japan

TEL: 81-6-6879-7446

Fax: 81-6-6879-4359

E-mail: [shimizu@ist.osaka-u.ac.jp](mailto:shimizu@ist.osaka-u.ac.jp)

**Table S1** Metabolite concentration obtained by CE-TOFMS (μmol g CDW^−1^)

| Strain | Kyokai 6 | | Kyokai 6 | | S288C | | Kyokai 6 | |
| --- | --- | --- | --- | --- | --- | --- | --- | --- |
| Methionine | − | | + | | + | | + | |
| Yeast extract  (g L^−1^) | 5.0 | | 5.0 | | 5.0 | | 0.0 | |
|  | Average | SD | Average | SD | Average | SD | Average | SD |
| Gly | 6.8 | 3.2 | 23.2 | 0.5 | 14.1 | 0.3 | 10.4 | 0.6 |
| Ala | 5.3 | 1.4 | 35.6 | 0.5 | 42.3 | 0.3 | 81.3 | 8.8 |
| Ser | 2.5 | 0.6 | 2.1 | 0.1 | 2.5 | 0.03 | 3.3 | 0.5 |
| Pro | 1.8 | 0.2 | 13.3 | 0.6 | 7.7 | 0.1 | 7.2 | 1.2 |
| Val | 28.9 | 6.3 | 9.0 | 0.1 | 12.8 | 0.2 | 4.1 | 0.8 |
| Thr | 3.6 | 1.4 | 6.4 | 0.2 | 6.4 | 0.1 | 7.6 | 1.0 |
| Cys | 0.6 | 0.1 | 0.3 | 0.04 | 0.6 | 0.02 | 0.1 | 0.04 |
| Ile | 1.3 | 0.8 | 3.0 | 0.1 | 4.3 | 0.1 | 2.1 | 0.5 |
| Leu | 2.3 | 0.7 | 3.8 | 0.2 | 6.6 | 0.2 | 1.3 | 0.4 |
| Asn | 6.6 | 1.2 | 5.7 | 0.1 | 5.1 | 0.1 | 5.6 | 0.9 |
| Asp | 5.2 | 0.5 | 5.0 | 0.2 | 2.4 | 0.04 | 2.8 | 0.6 |
| Gln | 28.8 | 4.5 | 3.0 | 0.04 | 10.8 | 0.6 | 6.7 | 0.9 |
| Lys | 30.8 | 5.4 | 23.6 | 0.6 | 43.9 | 0.8 | 18.9 | 2.3 |
| Glu | 80.9 | 0.9 | 49.8 | 3.3 | 61.7 | 3.5 | 46.0 | 7.0 |
| Met | 0.06 | 0.02 | 28.2 | 0.2 | 38.7 | 2.5 | 10.4 | 0.7 |
| His | 18.4 | 3.6 | 13.0 | 0.1 | 22.3 | 0.6 | 11.0 | 2.2 |
| Phe | 0.4 | 0.2 | 4.3 | 0.2 | 3.4 | 0.1 | 1.4 | 0.4 |
| Arg | 18.1 | 2.9 | 23.1 | 0.6 | 34.8 | 1.7 | 18.3 | 2.7 |
| Tyr | 3.9 | 1.8 | 3.9 | 0.1 | 4.6 | 0.2 | 3.1 | 0.7 |
| Trp | 0.4 | 0.1 | 0.9 | 0.1 | 0.8 | 0.1 | 0.5 | 0.1 |
| Ornithine | 1.8 | 0.4 | 2.8 | 0.3 | 3.7 | 0.4 | 3.6 | 0.3 |
| Citrulline | 0.3 | 0.1 | 0.03 | 0.001 | 0.1 | 0.01 | 0.2 | 0.02 |
| γ-Aminobutyric acid | 3.6 | 1.5 | 11.6 | 0.4 | 5.0 | 0.1 | 0.4 | 0.007 |
| Anthranilic acid | 0.003 | 0.004 | 0.01 | 0.0002 | 0.08 | 0.01 | 0.06 | 0.02 |
| Homoserine | 0.8 | 0.2 | 0.9 | 0.1 | 0.1 | 0.004 | 0.5 | 0.05 |

ND, not detected.

**Table S1** Metabolite concentration obtained by CE-TOFMS (μmol g CDW^−1^) (Continued)

| Strain | Kyokai 6 | | Kyokai 6 | | S288C | | Kyokai 6 | |
| --- | --- | --- | --- | --- | --- | --- | --- | --- |
| Methionine | − | | + | | + | | + | |
| Yeast extract  (g L^−1^) | 5.0 | | 5.0 | | 5.0 | | 0.0 | |
|  | Average | SD | Average | SD | Average | SD | Average | SD |
| Glucose 6-phosphate | 4.0 | 0.8 | 0.01 | 0.003 | 0.02 | 0.006 | 0.2 | 0.1 |
| Fructose 6-phosphate | 0.6 | 0.2 | 0.003 | 0.001 | 0.01 | 0.001 | 0.06 | 0.02 |
| Glucose 1-phosphate | 0.1 | 0.01 | 0.02 | 0.001 | 0.004 | 0.0005 | 0.04 | 0.006 |
| Fructose 1,6-diphosphate | 0.5 | 0.1 | ND | ‐ | 0.001 | 0.002 | 0.1 | 0.04 |
| Dihydroxyacetone phosphate | 0.1 | 0.0 | 0.007 | 0.01 | 0.003 | 0.003 | 0.10 | 0.03 |
| Glycerol 3-phosphate | 0.6 | 0.2 | 0.1 | 0.01 | 0.07 | 0.004 | 0.10 | 0.02 |
| 3-Phosphoglyceric acid | 2.7 | 0.7 | 0.1 | 0.02 | 0.2 | 0.03 | 0.2 | 0.03 |
| 2-Phosphoglyceric acid | 0.5 | 0.1 | 0.06 | 0.02 | 0.1 | 0.003 | 0.08 | 0.03 |
| Phosphoenolpyruvic acid | 1.0 | 0.3 | 0.09 | 0.02 | 0.2 | 0.04 | 0.08 | 0.04 |
| Acetyl CoA | 0.09 | 0.03 | ND | ‐ | 0.005 | 0.0002 | 0.009 | 0.002 |
| Citric acid | 26.1 | 6.3 | 4.4 | 0.4 | 1.7 | 0.215 | 10.3 | 3.2 |
| *cis*-Aconitic acid | 0.1 | 0.02 | 0.02 | 0.002 | 0.01 | 0.002 | 0.4 | 0.09 |
| Isocitric acid | 0.3 | 0.1 | 0.05 | 0.002 | 0.008 | 0.001 | 0.4 | 0.09 |
| 2-Oxoglutaric acid | 1.7 | 0.3 | 0.2 | 0.1 | 0.2 | 0.02 | 0.5 | 0.3 |
| Succinic acid | 7.7 | 1.6 | 35.6 | 2.7 | 32.2 | 1.1 | 23.0 | 4.0 |
| Fumalic acid | 3.7 | 0.5 | 0.2 | 0.007 | 0.8 | 0.09 | 0.7 | 0.2 |
| Malic acid | 14.1 | 2.1 | 1.1 | 0.05 | 5.1 | 0.3 | 5.5 | 1.8 |
| 6-Phosphogluconic acid | 0.06 | 0.03 | ND | ‐ | ND | ‐ | 0.02 | 0.004 |
| Ribulose 5-phosohate | 0.1 | 0.01 | 0.01 | 0.0007 | 0.01 | 0.002 | 0.09 | 0.03 |
| Sedoheptulose 7-phosohate | 0.4 | 0.1 | 0.0004 | 0.0007 | 0.004 | 0.002 | 0.02 | 0.004 |
| Choline | 0.04 | 0.01 | 0.4 | 0.1 | 0.06 | 0.01 | 0.5 | 0.05 |
| Betaine | 4.0 | 0.6 | 0.5 | 0.05 | 0.8 | 0.02 | 0.1 | 0.01 |
| Carnosine | 0.03 | 0.01 | 0.02 | 0.004 | 0.05 | 0.003 | 0.09 | 0.03 |
| Glutathione (GSSG) | 0.8 | 0.2 | 0.7 | 0.1 | 1.1 | 0.1 | 2.2 | 0.4 |
| Glutathionme (GSH) | 19.2 | 3.3 | 14.1 | 1.0 | 23.7 | 1.1 | 11.8 | 3.5 |

ND, not detected.

**Table S1** Metabolite concentration obtained by CE-TOFMS (μmol g CDW^−1^) (Continued)

| Strain | Kyokai 6 | | Kyokai 6 | | S288C | | Kyokai 6 | |
| --- | --- | --- | --- | --- | --- | --- | --- | --- |
| Methionine | − | | + | | + | | + | |
| Yeast extract  (g L^−1^) | 5.0 | | 5.0 | | 5.0 | | 0.0 | |
|  | Average | SD | Average | SD | Average | SD | Average | SD |
| Adenine | 0.007 | 0.002 | 0.1 | 0.01 | 0.02 | 0.004 | 0.2 | 0.05 |
| Uracil | 0.5 | 0.04 | 0.3 | 0.03 | 0.3 | 0.02 | 0.4 | 0.04 |
| Inosine | 0.1 | 0.05 | ND | ‐ | 0.01 | 0.001 | 0.1 | 0.03 |
| Adenosine | 0.07 | 0.01 | 0.3 | 0.004 | 0.09 | 0.008 | 0.04 | 0.01 |
| Guanosine | 0.1 | 0.04 | 0.01 | 0.01 | 0.004 | 0.0004 | 0.1 | 0.03 |
| Cytidine | 0.1 | 0.03 | 0.01 | 0.00 | 0.003 | 0.0002 | 0.007 | 0.003 |
| Uridine | 1.3 | 0.2 | 0.3 | 0.01 | 0.2 | 0.004 | 1.0 | 0.2 |
| Hypoxanthine | 0.04 | 0.02 | 0.1 | 0.01 | 0.06 | 0.002 | 0.1 | 0.009 |
| IMP | 0.004 | 0.001 | 0.1 | 0.01 | 0.06 | 0.004 | 0.4 | 0.1 |
| AMP | 0.1 | 0.03 | 2.3 | 0.2 | 0.5 | 0.04 | 0.4 | 0.2 |
| GMP | 0.01 | 0.002 | 1.6 | 0.1 | 0.4 | 0.04 | 0.2 | 0.1 |
| CMP | 0.02 | 0.02 | 1.1 | 0.1 | 0.5 | 0.04 | 0.06 | 0.04 |
| UMP | 0.02 | 0.02 | 3.1 | 0.2 | 0.8 | 0.07 | 0.2 | 0.1 |
| ADP | 2.3 | 0.6 | 0.4 | 0.1 | 0.3 | 0.05 | 0.7 | 0.4 |
| GDP | 0.2 | 0.1 | 0.3 | 0.03 | 0.3 | 0.04 | 0.5 | 0.3 |
| CDP | 0.1 | 0.02 | 0.1 | 0.01 | 0.08 | 0.01 | 0.1 | 0.05 |
| UDP | 0.2 | 0.04 | 0.2 | 0.05 | 0.4 | 0.08 | 0.4 | 0.2 |
| ATP | 13.6 | 1.8 | 0.02 | 0.01 | 0.2 | 0.04 | 0.8 | 0.3 |
| GTP | 1.2 | 0.3 | 0.01 | 0.003 | 0.2 | 0.03 | 0.5 | 0.2 |
| CTP | 1.2 | 0.3 | 0.002 | 0.003 | 0.08 | 0.01 | 0.1 | 0.04 |
| UTP | 2.1 | 0.6 | 0.009 | 0.003 | 0.3 | 0.04 | 0.6 | 0.2 |
| Glycolic acid | 0.3 | 0.1 | 0.3 | 0.1 | 0.4 | 0.1 | 0.02 | 0.04 |
| 3-hydroxybutiric acid | 0.7 | 0.03 | 0.04 | 0.009 | 0.04 | 0.001 | 0.2 | 0.02 |
| 2-Oxoisovaleric acid | 0.1 | 0.03 | ND | ‐ | ND | ‐ | 0.04 | 0.006 |
| Gluconic acid | 0.1 | 0.01 | 0.1 | 0.008 | 0.03 | 0.001 | 0.2 | 0.05 |

ND, not detected.

**Table S2** Factor loading corresponding to the PC1 and PC2.

|  | Factor loadings | |
| --- | --- | --- |
| Metabolite | PC1 | PC2 |
| Gly | 2.71 | 0.49 |
| Ala | 2.94 | -0.60 |
| Ser | -0.50 | -1.84 |
| Pro | 2.97 | 0.44 |
| Val | -2.82 | -0.67 |
| Thr | 2.56 | -0.56 |
| Cys | -1.67 | -2.41 |
| Ile | 2.48 | -1.23 |
| Leu | 2.29 | -1.86 |
| Asn | -1.69 | 0.75 |
| Asp | -1.23 | 2.58 |
| Gln | -2.63 | -1.43 |
| Lys | -0.10 | -2.71 |
| Glu | -2.78 | -0.97 |
| Met | 2.96 | -0.47 |
| His | -0.62 | -2.84 |
| Phe | 2.97 | -0.18 |
| Arg | 2.10 | -2.08 |
| Tyr | 0.96 | -1.22 |
| Trp | 2.74 | -0.07 |
| Ornithine | 2.48 | -1.49 |
| Citrulline | -2.67 | -1.31 |
| γ-Aminobutyric acid | 2.35 | 1.30 |
| Anthranilic acid | 1.63 | -2.02 |
| Homoserine | -0.96 | 2.79 |

**Table S2** Factor loading corresponding to the PC1 and PC2. (Continued)

|  | Factor loadings | |
| --- | --- | --- |
| Metabolite | PC1 | PC2 |
| Glucose 6-phosphate | -2.97 | 0.20 |
| Fructose 6-phosphate | -2.97 | -0.26 |
| Glucose 1-phosphate | -2.59 | 1.44 |
| Fructose 1,6-diphosphate | -2.96 | 0.14 |
| Dihydroxyacetone phosphate | -2.65 | 0.21 |
| Glycerol 3-phosphate | -2.71 | 0.66 |
| 3-Phosphoglyceric acid | -2.99 | 0.00 |
| 2-Phosphoglyceric acid | -2.96 | -0.16 |
| Phosphoenolpyruvic acid | -2.95 | -0.24 |
| Acetyl CoA | -2.86 | -0.81 |
| Citric acid | -2.61 | 1.25 |
| *cis*-Aconitic acid | -2.86 | 0.70 |
| Isocitric acid | -2.34 | 1.84 |
| 2-Oxoglutaric acid | -2.97 | 0.07 |
| Succinic acid | 2.98 | -0.24 |
| Fumalic acid | -2.77 | -1.15 |
| Malic acid | -2.59 | -1.51 |
| 6-Phosphogluconic acid | -2.97 | 0.37 |
| Ribulose 5-phosohate | -2.97 | 0.30 |
| Sedoheptulose 7-phosohate | -2.97 | -0.17 |
| Choline | 2.14 | 2.02 |
| Betaine | -2.95 | -0.27 |
| Carnosine | -0.16 | -2.81 |
| Glutathione (GSSG) | 0.08 | -2.55 |
| Glutathionme (GSH) | -0.49 | -2.86 |

**Table S2** Factor loading corresponding to the PC1 and PC2. (Continued)

|  | Factor loadings | |
| --- | --- | --- |
| Metabolite | PC1 | PC2 |
| Adenine | 2.55 | 1.45 |
| Uracil | -2.74 | 0.57 |
| Inosine | -2.66 | -1.35 |
| Adenosine | 2.22 | 1.91 |
| Guanosine | -2.63 | 1.17 |
| Cytidine | -2.56 | 1.38 |
| Uridine | -2.76 | 0.96 |
| Hypoxanthine | 2.47 | -0.07 |
| IMP | 2.97 | 0.35 |
| AMP | 2.77 | 1.08 |
| GMP | 2.97 | 0.40 |
| CMP | 2.81 | -0.11 |
| UMP | 2.88 | 0.09 |
| ADP | -2.83 | 0.55 |
| GDP | 2.47 | -0.74 |
| CDP | -0.72 | -1.20 |
| UDP | 2.02 | -1.97 |
| ATP | -2.91 | -0.70 |
| GTP | -2.58 | -1.51 |
| CTP | -2.58 | -1.45 |
| UTP | -2.54 | -1.58 |
| Glycolic acid | -0.16 | -1.37 |
| 3-hydroxybutiric acid | -2.94 | 0.43 |
| 2-Oxoisovaleric acid | -2.93 | 0.26 |
| Gluconic acid | -0.92 | 2.60 |

**Table S3** Fermentation profiles of Kyokai 6 strains in the presence of glucose with 1.5 g L^−1^ l-methionine

|  |  | Yeast extract (g L^−1^) | | | |
| --- | --- | --- | --- | --- | --- |
|  |  | 0.0 | 1.25 | 2.5 | 5.0 |
| SAM production (mg L^−1^) |  | 315.6 ± 21.4 | 243.0 ± 2.7 | 218.7 ± 2.1 | 189.8 ± 6.4 |
| Biomass (g CDW L^−1^) |  | 3.7 ± 0.1 | 4.8 ± 0.03 | 5.1 ± 0.02 | 5.7 ± 0.03 |
| SAM content  (mg g CDW^−1^) |  | 86.1 ± 3.2 | 50.4 ± 0.7 | 43.2 ± 0.6 | 33.6 ± 0.9 |
| Ethanol (g L^−1^) |  | 18.6 ± 0.1 | 19.0 ± 0.2 | 19.2 ± 0.4 | 19.3 ± 0.04 |
| pH |  | 4.6 ± 0.04 | 5.1 ± 0.1 | 5.2 ± 0.02 | 5.4 ± 0.01 |

All data were obtained from the averages of three independent experiments ± S.D.
